# Virtual Consultations and the Role of Technology During the COVID-19 Pandemic for People With Type 2 Diabetes: The UK Perspective

**DOI:** 10.2196/21609

**Published:** 2020-08-28

**Authors:** Lauren M Quinn, Melanie J Davies, Michelle Hadjiconstantinou

**Affiliations:** 1 Leicester Diabetes Centre University of Leicester Leicester United Kingdom; 2 Leicester Diabetes Research Centre University of Leicester Leicester United Kingdom

**Keywords:** diabetes, virtual clinic, technology, COVID-19, United Kingdom, pandemic, feasibility, effective, telehealth

## Abstract

The coronavirus disease (COVID-19) pandemic has presented unique challenges for people with diabetes, in addition to their high-risk stratification for infection. Supporting people with diabetes to self-care has been critical to reduce their risk of severe infection. This global pandemic has presented an opportunity to digitalize diabetes care and rapidly implement virtual diabetes clinics, with the aim of optimizing diabetes management and well-being, while keeping patients safe. We performed a rapid review of the literature to evaluate the feasibility and effectiveness of virtual clinics in diabetes care before and during the COVID-19 pandemic and have combined these findings with our own reflections in practice. We identified examples demonstrating safety and feasibility of virtual diabetes clinics, which aligns with our own clinical experience during the pandemic. The advantages of virtual clinics include reduced treatment burden, improved therapeutic alliances, societal and psychological benefits, and in our experience, innovative solutions to overcome the challenges presented by the transition from in-person to virtual care. We have provided three infographics to illustrate lessons learned and key recommendations, including steps to establish a virtual diabetes clinic, a checklist guide for health care professionals conducting virtual clinics, and a patient guide for making the most out of the virtual clinic. It is important to continue adapting to this pandemic and to make technology a sustainable option for the future of diabetes care.

## Introduction

We are in the midst of the coronavirus disease (COVID-19) pandemic, caused by severe acute respiratory syndrome coronavirus 2 (SARS-CoV-2), which has resulted in thousands of deaths worldwide [1,2]. Increased age and underlying health conditions, including diabetes, cardiovascular disease, obesity, and hypertension, significantly increase the risk of COVID-19 infection [3,4]. Similarly, disease severity may be worsened, and deaths are overrepresented in people with diabetes [4,5]. Evidence shows poor glycemic control is both associated with and a consequence of COVID-19 infection, the latter demonstrated in older persons with type 2 diabetes [3,5,6]. More recently, evidence has shown that Black, Asian, and minority ethnic groups are more severely affected, with higher death rates observed from COVID-19 infection in this population; high prevalence of diabetes and comorbidities in this subgroup likely contributes to this increased risk [7]. Despite the challenges for people with diabetes, the COVID-19 pandemic has presented a valuable opportunity to digitalize diabetes care. Given the importance of maintaining and improving well-being and glycemic control during this time, evaluation of novel methods to support self-management remotely is critically important.

The aims of this paper are (1) to explore the evidence for the role of telemedicine to support people with diabetes during the COVID-19 pandemic and beyond; (2) to outline the benefits and challenges presented by virtual diabetes care; (3) to present our experience of virtual consultations in clinical settings during the COVID-19 pandemic; and (4) to share lessons learned to assist researchers, clinicians, and people with diabetes when integrating technology in diabetes care.

## Impact of Social Distancing/Shielding on Diabetes Care and Well-Being

With diabetes being classed as a high-risk group by the government, it is important that people with diabetes take care of their health now more than ever. People with diabetes are advised to practice social distancing (eg, working from home or self-isolating), adhere to national recommendations on frequent handwashing, and abstain from nonessential travel to avoid infectious contacts [8]. In some cases, extremely vulnerable people with diabetes are advised to undertake “shielding.” Additional guidance, specifically for people with diabetes, focuses on self-management strategies, which help to boost innate immunity for primary prevention [8,9]. The American Association of Diabetes Educators recommends the following seven self-care activities: keeping physically active, healthy diet, following medication regimen, blood glucose monitoring and problem solving, reducing risk of complications, and self-empathy; all of these are endorsed for people with diabetes to reduce their risk and severity of COVID-19 [10].

Interestingly, for people with diabetes, their engagement with these self-care activities has been highly variable during the pandemic, with some being able to focus more time on their diabetes management and undertake increasing amounts of self-care. For example, Bonora et al [11] showed in a small study that glycemic control improved for people with type 1 diabetes during lockdown who self-isolated compared to those who continued working [11]. However, for others, the significant change in lifestyle presented by lockdown has been detrimental to their health and well-being. Barriers specifically for people with diabetes have included difficulty accessing healthy foods because of restricted shopping and bulk buying; inability to access medications; restricting physical activity to once per day in the local area; and being unable to attend face-to-face appointments with their diabetes care providers [8,9]. With this in mind, diabetes health care professionals (HCPs) have valid concerns that glycemic control, quality of life, self-management, and well-being can be significantly jeopardized during social distancing and shielding, posing considerable risks to people with diabetes, both in the short and long term. Lockdown, social distancing, shielding, and the abundance of misinformation in the media also present additional stressors, which may further exacerbate underlying depression and anxiety [12], conditions that are already highly prevalent in the diabetes population [13].

## Digitalization of Diabetes Care: Benefits and Challenges

### Digital Consultations

Technology has been increasingly integrated into diabetes education and care in modern times, for example, through apps, computer-based or web-based education, and telemedicine [14]. The NHS (National Health Service) long-term plan set out to increase digitalization within NHS programs [15,16]. This included roll-out of virtual or non–face-to-face clinics, with the aim of reducing face-to-face appointments by one third over the next 5 years [16]. This non–face-to-face activity may be synchronous or asynchronous, meaning a direct or indirect line of communication with an HCP, respectively [17,18]. Synchronous activity would be a video or telephone consultation, whereas asynchronous may involve monitoring an email or tracking system and responding to patients’ questions through these platforms. The benefits of non–face-to-face appointments are multifactorial and include the opportunity for better care and more connected patient care pathways, as well as cost savings and a reduced environmental impact.

Studies evaluating virtual clinics prior to the COVID-19 pandemic have demonstrated feasibility, accessibility, safety, and effectiveness comparable to in-person consultations [19]. A multicenter mixed methods study evaluating video consultations in diabetes care showed that video consultations were shorter in duration and people with diabetes did relatively more talking than the HCP [20,21]. Although from a management perspective, video consultations were favored, there were significant barriers to uptake from the teams implementing them because of the significant changes introduced to their usual way of working and the care processes, systems, and pathways [21]. A Cochrane review analyzed 21 low-to-high-quality studies comparing telemedicine to usual care in people with diabetes and found that improvement in glycemic control was variable, but low-density lipoprotein (LDL) levels and blood pressure were more effectively lowered by telemedicine approaches compared to usual care [22]. Further studies have evaluated virtual clinics for diabetes care in type 1 and type 2 diabetes and have reported improved biochemical parameters, including glycated hemoglobin (HbA_1c_) [23]. However, many of these studies combined interventions, such as synchronous and asynchronous programs, rendering it difficult to delineate the efficacy of the individual interventions [23]. Studies focusing on synchronous video consultations have been of short duration with a limited sample size [24]. Additionally, how these findings relate to virtual clinics being implemented during and due to a global pandemic must be considered.

During the COVID-19 pandemic, telemedicine has been widely adopted globally in order to reduce exposure and need for people with diabetes to come into a hospital, while maintaining care standards for people with chronic conditions. For example, virtual consultations have been implemented to triage patients suspected of COVID-19 in primary care and to initiate a hospital visit [25-27]. There have been other examples of successful virtual care adoption for people with newly diagnosed type 1 diabetes [28], and for people with long-standing type 1 diabetes. A small Italian study showed that for people with type 1 diabetes who were not working during the pandemic and using continuous glucose monitoring, their time in range significantly improved from 54% to 65%, and this was attributed to decreased hyperglycemia [11]. Blood glucose variability and average glucose readings from continuous glucose monitoring also significantly improved in this study. In contrast to patients who continued working, there was no difference in glycemic control [11]. Early reports suggest that virtual clinics are feasible, with some centers increasing virtual clinic consultations from 1% of all consultations prior to COVID-19 to 70% afterwards, and it is technology that has made this rapid transformation possible [29-31].

A recent linguistic ethnographic study has shown that video consultations among people with a long-term condition and their clinician was found to be effective [26]. When patients experienced technical or operational issues with their video equipment or internet connection, they generally found a solution to resolve the problem. Familiarity and experience with technology helped in situations like these. Technical interruptions and delays of connectivity either on patient or clinician device were clearly evident in the study; nevertheless, remote physical examinations were conducted, allowing the patient and/or carer to take an active role in the consultation [26].

### Benefits of Virtual Clinics

#### Patient Safety

In line with social distancing advice and minimizing risk for people with diabetes, virtual clinics bring specialist care to the home of the patient. The virtual clinic is the ideal solution to enable patients to access specialist care but without unnecessarily exposing themselves to a high-risk environment, or come in close contact with staff or other patients. This is particularly pertinent given the new data supporting the number of COVID-19 infections developed while being in hospital [32].

#### Reduced Burden of Treatment: Improved Accessibility and Overall Experience

Virtual clinics increase accessibility to specialist diabetes care. For example, those shielding can still attend a virtual appointment, those working can more easily take the time out from their day (eg, over a coffee break), and those who would normally be unable to travel would no longer need to. In our experience, we have found that patients who regularly did not attend appointments are now attending their virtual clinic appointments. The virtual clinic may also provide an improved patient experience because people with diabetes are not required to spend additional time traveling to the hospital for regular appointments, which can significantly reduce the burden of treatment that some may experience with their diabetes care. The virtual clinics may provide a solution—video or phone calls may provide a relaxed environment for some people with diabetes, as they are within their own familiar surroundings, which may create a more relaxed atmosphere for their virtual appointment. From our clinic team experience of using virtual consultations with people with diabetes, it is evident that some patients feel comfortable showing the HCP their house environment and exercise regimen, and introduce them to their family members, within professional boundaries. This offers a unique insight into environmental or interpersonal factors, which may influence the self-care activities of people with diabetes. Long-term, these factors may be considered when discussing the care plan and treatment goals of people with diabetes. The opportunities presented in virtual clinics for delivering increasingly person-centered and individualized care cannot be underestimated.

#### Improved Therapeutic Alliance and Consultation Dynamics

**Figure 1:**
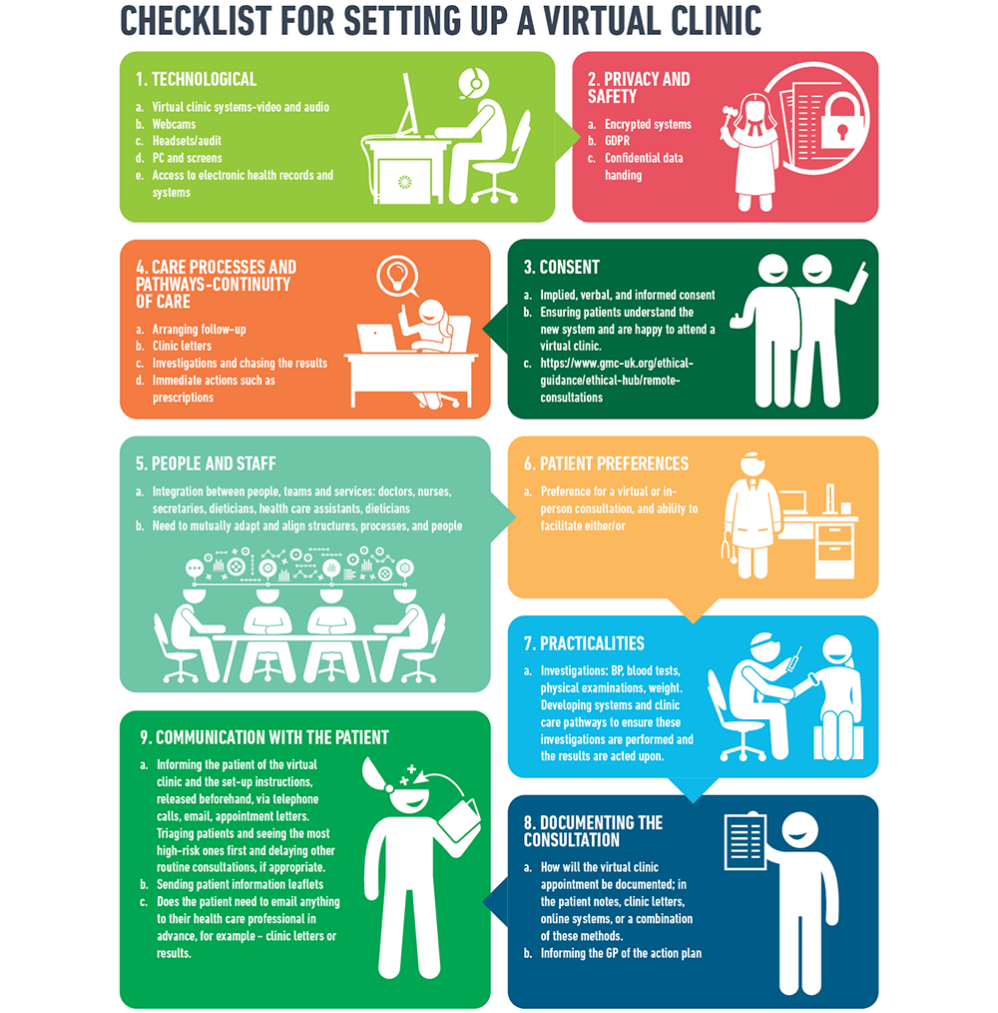
Checklist for setting up a virtual clinic. GDPR: General Data Protection Regulation; BP: blood pressure; GP: general practitioner.

Virtual consultations could significantly shift the locus of control of people with diabetes, which would complement the philosophy around the importance of self-management and self-care in diabetes. Virtual consultations could provide an opportunity for shared care between people with diabetes and the HCP. Multiple members of the diabetes multidisciplinary team (MDT) may come together for the video consultation, to make collaborative decisions with patients. From our experience, this results in a more effective consultation. People with diabetes benefit from MDT input, with reduced time commitment for patients and professionals alike. Physical examination (eg, conducting a diabetes foot examination) can also be performed. This is particularly important in the context of diabetes foot disease, where people with diabetes can be triaged in virtual clinic to determine the need for hospital admission based on the video examination findings. Technical barriers would be expected when setting up virtual consultations; thus, we have shared a checklist that we developed for setting up a virtual clinic (Figure 1).

#### Societal and Psychosocial Benefits

Virtual clinics reduce the need for travel to and from hospitals, which is a significant benefit since 20% of traffic in the United Kingdom is attributed to health care–related travel [15,16]. Additionally, virtual clinics mean less time is missed from work, which can be a recurrent issue for people with diabetes, so this reduction in treatment burden is highly advantageous.

With social isolation becoming more prevalent than ever during the current climate, it is imperative to acknowledge the positive impact that remote consultations may have on individuals who are required to practice social distancing and shielding. Virtual consultations, whether these are conducted by telephone or video, provide people with diabetes the opportunity to virtually connect with their HCP, mitigating the psychological effects of social isolation.

### Overcoming the Challenges of Virtual Care

Elements of the routine diabetes clinic (eg, checking blood pressure, sampling the urine, HbA_1c_, spot checks, and physical examination) are not possible in the same way in a virtual consultation. However, given the importance of these methods to screen for diabetes complications, alternative strategies can be developed to make them possible. For example, people with diabetes can have their own blood pressure monitor. This may lead to more accurate readings, with the absence of white coat hypertension. Also, urine samples can be delivered locally to general practices and examination can still be performed via video consultation. For example, diabetes foot disease can be screened for and people with diabetes can be triaged to determine need for further assessment.

#### Patient Preference

People with diabetes can choose whether to have a telephone or video consultation, to reflect variability in access to technology and systems for video recording and to ensure access to care services. However, virtual care is a significant change from the usual hospital attendance for their diabetes care, thus, some people with diabetes would still prefer a face-to-face consultation. However, in light of the COVID-19 pandemic, people with diabetes have readily transitioned to virtual methods, and it is hoped that through familiarization with these new consultation methods, this may increase uptake of virtual care in the longer term. There are also concerns that people with diabetes may find it difficult to build rapport with their HCP in a virtual clinic, but in our experience, the opposite is true. People with diabetes have been far more relaxed in the virtual clinic and able to speak more openly with their diabetes team, which is essential to improve their diabetes management. But this is dependent on high-quality video image and sound, highlighting the essential role of technology to optimize communication between people with diabetes and their HCP.

#### Engaging the Multidisciplinary Team

**Figure 2:**
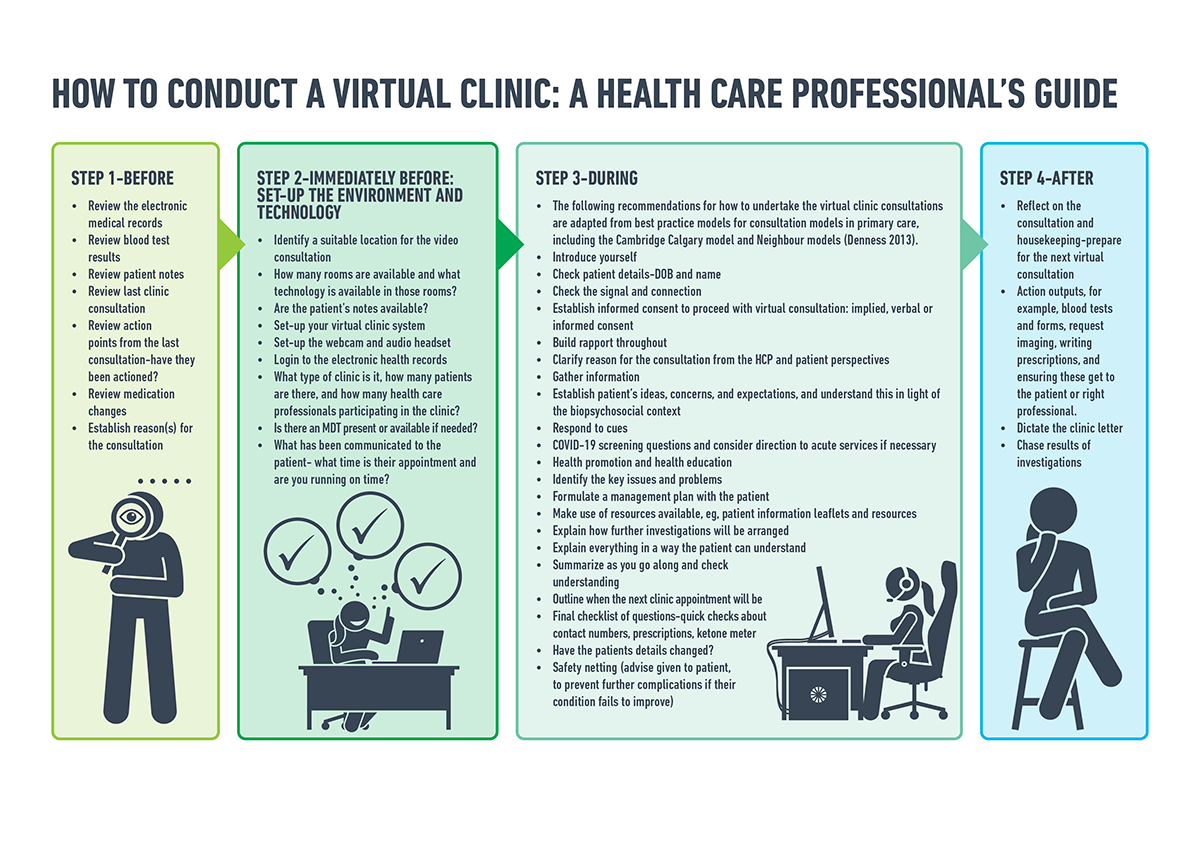
How to conduct a virtual clinic: a health care professional's guide. MDT: multidisciplinary team; DOB: date of birth; HCP: health care professional; COVID-19: coronavirus disease.

Engaging professionals and the wider team with virtual care can be a challenge in itself. This is a significant change for the diabetes team as well as the patient, and new processes and pathways have needed to develop rapidly to manage the care of people with diabetes remotely (Figure 2 [33]). For example, the ways in which clinic outcomes are actioned have changed and the wider clinical and nonclinical teams have needed to collaborate to generate these new systems. It is essential that the whole team is committed to the virtual care approach, because this ensures the delivery of safe, high-quality care. It is anticipated that with increased familiarity and established systems, HCPs will be more open to adopt virtual care into their routine practice and overcome the natural aversion to change of how it has always been.

### The Future of Virtual Clinics

Anecdotally, in our experience virtual clinics have been feasible and accessible, with high patient satisfaction. Virtual clinical consultations offer a different kind of benefit compared to conventional face-to-face appointments, particularly around convenience, logistics, cost-effectiveness, and clinician-patient dynamic/relationship [26]. However, we must acknowledge the pitfalls of these new modes of communication and the challenges that may lie ahead with the clinical quality and safety of appointments.

Building on these developments, we are looking to make virtual clinics sustainable for the long term. This is in line with the NHS long-term plan to make better use of data and digital technology in the next 5 years [15,16]. In a time of a pandemic, individualized care is more important than ever and virtual clinics provide a readily accessible solution to facilitate this. Having applied virtual clinics in our setting for the last 2 months, lessons we have learned include: (a) the importance of integrating multiple members of the MDT into the one virtual consultation; (b) avoiding the checklist approach and instead focusing on an individualized, person-centered consultation; (c) and acknowledging that video consultation may be preferred to telephone because of the additional benefits of human contact, body language, and the opportunity to gain better insight into the lifestyle and livelihood of people with diabetes in order to tailor medical support accordingly. In our experience, virtual clinics may be better suited to individuals with longstanding diabetes and where possible should be performed by a professional with whom they have already built a strong rapport.

Although virtual clinics can be an alternative option, there are key elements that must be considered to make the consultation as efficient as possible. Firstly, even though virtual consultations would require less resources compared to a face-to-face visit, organizational factors prior to the virtual consultation would still be required in order to book and record a clinic appointment. Secondly, logistical and administrative factors must be integrated within the NHS system, a system which for so many years has been based on delivering face-to-face patient services. We are programmed to deliver our outpatient services in a “traditional” way, and therefore would anticipate a colossal challenge adapting this existing pathway to a more digital-focused platform.

**Figure 3:**
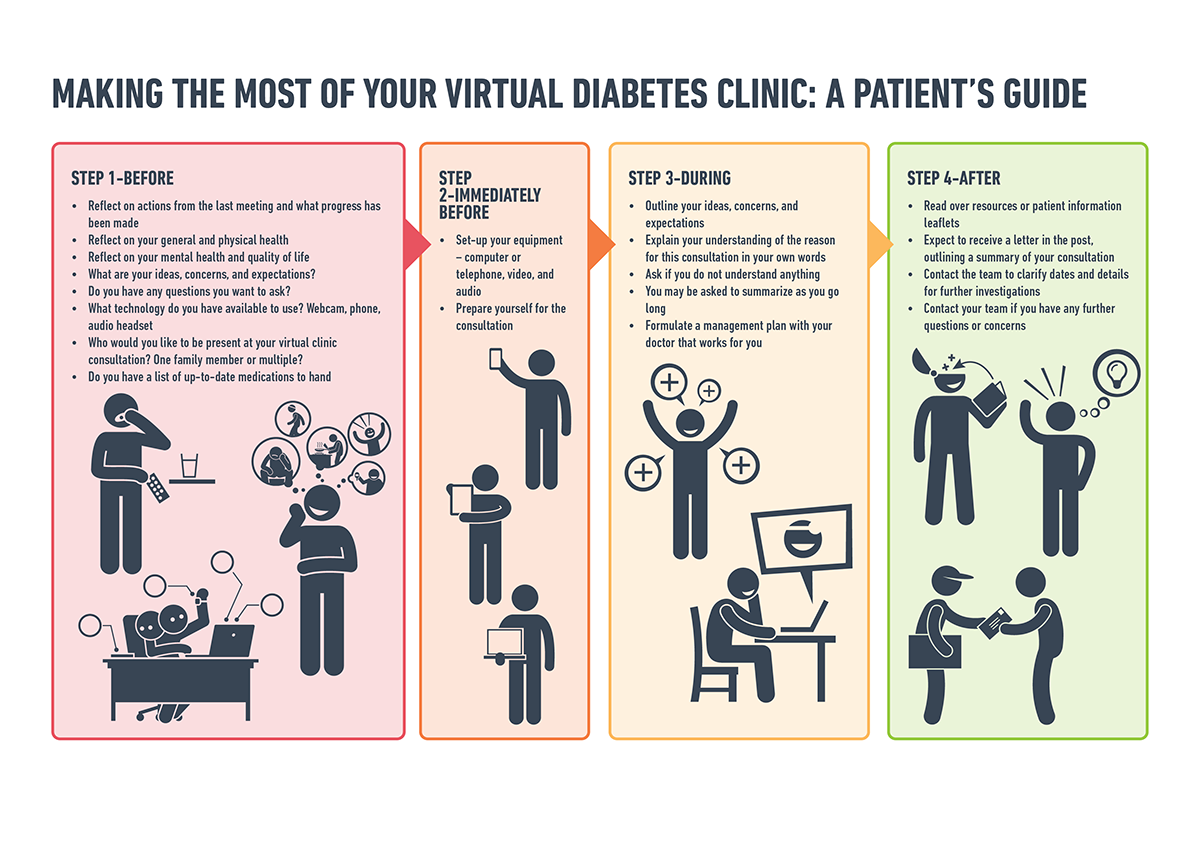
Making the most of your virtual diabetes clinic: a patient's guide.

By acknowledging the complexity of integrating virtual consultations, we also acknowledge the challenges that may come with technology, in terms of the security and safety of every patient and HCP. Data protection and privacy is of critical importance; the technology, software, and programs used in virtual clinics must be encrypted and adhere to the General Data Protection Regulation and information governance standards to maintain patient confidentiality at all times. We acknowledge that virtual consultations are not for everyone; however, providing options to people enables them to choose an approach that is tailored to their diabetes needs and lifestyle demands, with the aim of reducing the burden of treatment that so many people with diabetes may be experiencing. We aim to not only prepare our patients to attend virtual consultations, by creating a safe environment and respecting their safety (Figure 3), but to also create a digital platform that would integrate within the current NHS system.

## Conclusion

Virtual consultations may become a necessity following this pandemic. The current system pressures due to COVID-19 have led to numerous challenges to the delivery of routine diabetes care and education. Despite the relative lack of data to support virtual care, in the face of adversity, these virtual measures have been imperative to maintain a line of communication with people with diabetes and to support self-management and self-care remotely. With the right infrastructure and systems in place, technology is the key to evolution in diabetes care, and virtual consultations can be effectively embedded into routine diabetes care at the national level in the United Kingdom. At present, virtual clinics may be an ideal platform to reduce social isolation, encourage self-management remotely and in a less intrusive manner, and reduce burden of treatment. The COVID-19 outbreak will shift the culture of health care across the world and the way we interact within clinical settings will gradually change to ensure that care can be delivered within social distancing rules

## Abbreviations

COVID-19: coronavirus disease
HbA_1c_: glycated hemoglobin
HCP: health care professional
LDL: low-density lipoprotein
MDT: multidisciplinary team
NHS: National Health Service
SARS-CoV-2: severe acute respiratory syndrome coronavirus 2
